# Comparative Analysis of the Genetic Basis of Branched Nonylphenol Degradation by *Sphingobium amiense* DSM 16289^T^ and *Sphingobium cloacae* JCM 10874^T^

**DOI:** 10.1264/jsme2.ME18077

**Published:** 2018-12-05

**Authors:** Mina Ootsuka, Tomoyasu Nishizawa, Morifumi Hasegawa, Yasurou Kurusu, Hiroyuki Ohta

**Affiliations:** 1 United Graduate School of Agricultural Science, Tokyo University of Agriculture and Technology 3–5–8 Saiwai-cho, Fuchu-shi, Tokyo 183–8509 Japan; 2 Ibaraki University College of Agriculture 3–21–1 Chuo, Ami-machi, Ibaraki 300–0393 Japan

**Keywords:** flavin-dependent monooxygenase, *opdA*, *nmoA*, genome analysis, gas chromatography–mass spectrometry

## Abstract

Branched nonylphenol (BNP), a degradation product of nonylphenol polyethoxylates, exerts estrogenic effects on various organisms. The genes underlying BNP degradation by *Sphingobium amiense* DSM 16289^T^ were analyzed by complete genome sequencing and compared with those of the versatile BNP-degrading *Sphingobium cloacae* JCM 10874^T^. An *opdA* homolog (*opdA*_DSM16289_) encoding BNP degradation activity was identified in DSM 16289^T^, in contrast with JCM 10874^T^, possessing both the *opdA* homolog and *nmoA*. The degradation profile of different BNP isomers was examined by *Escherichia coli* transformants harboring *opdA*_DSM16289_, *opdA*_JCM10874_, and *nmoA*_JCM10874_ to characterize and compare the expression activities of these genes.

Branched nonylphenol (BNP) is a persistent compound known to exert estrogenic effects in aquatic organisms, mammals, and birds (23, 30, 37). The environmental occurrence of BNP is mainly due to the biodegradation of nonylphenol polyethoxylates (NPEOx), which are non-ionic surfactants used widely in industrial and agricultural applications (16, 24). This estrogenic activity is found in the shorter homologs of NPEOx, as well as BNP, but not in the higher homologs. Bacteria that degrade NPEOx are known to be ubiquitous in various environments (2, 16, 24). Commercial NPEOx is produced by the ethoxylation of technical-grade BNP, which is composed of at least 22 *para*-isomers possessing different degrees of branching in their alkyl side chain (36), and, thus, environmentally occurring BNP is recalcitrant to biodegradation. Previous studies demonstrated that BNP isomers have different biodegradabilities and estrogenicities in the environment (for a review, see ref. 23). The isolation of a bacterial strain capable of utilizing BNP as the sole carbon and energy source was pioneered by Tanghe *et al.* (32) in 1999, and the strain was designated as *Sphingomonas* sp. TTNP3. Following that study, several BNP-degrading bacteria were isolated, most of which were characterized phylogenetically as members of the *Sphingomonas/Sphingobium* group: *Sphingobium cloacae* S-3^T^ (=JCM 10874^T^) (7), *Sphingobium amiense* YT^T^ (=DSM 16289^T^) (6, 34), *Sphingobium xenophagum* Bayram (8), and *Sphingomonas* sp. NP5 (31). Among these BNP-degrading bacteria, the strains TTNP3 and Bayram were studied in-depth for their BNP degradation pathway, and the initial step of degradation was found to be a type II *ipso* substitution mechanism (5, 9, 10, 18). In this mechanism, BNP is oxidized at the aromatic carbon atom, followed by substitution with an alkyl side chain, resulting in separation to a hydroquinone and carbocation with nine carbon atoms. The enzymes involved in the type II *ipso* substitution of BNP and BNP-related octylphenol (OP) were identified as the flavin-dependent monooxygenase (14) octylphenol 4-monooxygenase (OpdA), which was initially identified in OP-degrading *Sphingomonas* sp. PWE1 (27). This enzyme was subsequently found in the BNP-degrading strains TTNP3 and Bayram (28), suggesting that OpdA is also responsible for BNP degradation. Thereafter, it was revealed that BNP-degrading *Sphingomonas* sp. NP5 had two identical nonylphenol monooxygenase genes (*nmoA*) and their predicted protein sequence showed 83% identity to the OpdA of *Sphingomonas* sp. PWE1 (31).

A high-resolution gas chromatographic analysis of BNP achieved the resolution of 18 *para*-isomers, which were classified into five distinct groups (36). By using this method, we previously demonstrated that BNP isomers with the α-dimethyl, α-methyl and α-ethyl structures (groups 1 and 2, respectively, in the left side columns of Table 1) were rapidly degraded by strain DSM 16289^T^ (initial degradation rate, approx. 0.43 h^−1^ for 10 mg L^−1^ BNP), while the breakdown of isomers with highly branched chains (groups 3–5 in Table 1) was markedly less efficient (15). In contrast, strain JCM 10874^T^ showed almost no substrate preference and degraded all the BNP isomers at similar rates (initial degradation rate, approx. 1.04 h^−1^ for 10 mg L^−1^ BNP). Prior to this study, we identified an *opdA* homolog and the *nmoA* gene in the two separate circular plasmids of strain JCM 10874^T^ using a genome analysis (26). In the present study, to clarify the genetic background of BNP degradation by the strain DSM 16289^T^, we elucidated its complete genome sequence and identified an *opdA* homolog in a circular plasmid. Furthermore, to clarify differences in their preferences for BNP isomers, we compared the degradation profile of BNP isomers by three *Escherichia coli* transformants harboring the *opdA* homologs of the two strains and the *nmoA* gene of strain JCM 10874^T^.

The strains DSM 16289^T^ and JCM 10874^T^ were obtained from the DSMZ culture collection in Germany and RIKEN BRC-JCM in Japan, respectively. These strains were grown at 30°C in two-fold (strain DSM 16289^T^) and five-fold (strain JCM 10874^T^) diluted nutrient broth (pH 7.2). Nutrient broth comprised 1.0% (w/v) Bonito extract (Wako Pure Chemical Industry, Osaka, Japan), 1.0% (w/v) Bacto Peptone (Beckton, Dickinson and Company, Sparks, MD, USA), and 0.5% (w/v) NaCl. Genomic DNA was extracted from the cultured cells as described previously (25), and the sequencing of genomic DNA from the strain DSM 16289^T^ was performed using PacBio RS II and the HiSeq 2500 instrument at GeneBay, Yokohama, Japan (http://genebay.co.jp). The hybrid assembly with PacBio RSII and HiSeq 2500 was performed by SPAdes v.3.10.1 (4), Unicycler v.0.4.0 (38), and Canu v.1.5 (20). Genome sequences were annotated using the Microbial Genome Annotation Pipeline (MiGAP: https://www.migap.org), followed by manual annotation with the NCBI-nr databases using the BLASTP program (3). tRNA was predicted using tRNAscan-SE 2.0 (22), and GC contents were calculated using GC-Profil (11). Multiple sequence alignments and a phylogenetic analysis using the UPGMA method were performed with Clustal W (33) and MEGA v.7.0.26 (21), respectively.

The *opdA* homologs of the strains DSM 16289^T^ and JCM 10874^T^ were amplified by PCR using the opdA-forward primer (5′-TTC ATC CTG AAA GAC ACT GCC GGA-3′) and opdA-reverse primer (5′-ACG CGC TTC CAG ACC AAC CTA TTT-3′) (27). The primers for *nmoA* gene amplification were originally designed from the *opdA* sequence (27) as forward: 5′-ATC CAA CGC GAA CAA CTT CGT GCA-3′ (nmoA-F) and reverse: 5′-TCA TTA TGG CTA GCG CGC CTA CTT-3′ (nmoA-R). PCR was performed using KOD-Plus-Neo (TOYOBO, Osaka, Japan) under the following conditions: 94°C for 2 min and 25 cycles of 98°C for 10 s, 72°C for 30 s, and 68°C for 50 s. Amplicon products were subcloned into pGEM-T Easy plasmid vectors (Promega, Madison, WI, USA), and the resulting plasmids were transformed into *E. coli* JM 109 (Takara Bio, Shiga, Japan). The constructed plasmids were designated pSA*opdA*, pSC*opdA*, and pSC*nmoA* for the *opdA* homologs of strains DSM 16289^T^ and JCM 10874^T^ and the *nmoA* gene of strain JCM 10874^T^, respectively. The transformants were grown on agar plates of LB (29) medium containing 100 μg mL^−1^ ampicillin (LB/Amp) and 200 mg L^−1^ BNP (Kanto Chemical, Tokyo, Japan). Aliquots of 100 mM IPTG aqueous solution (100 μL) and 40 mg mL^−1^ X-gal (5-bromo-4-chloro-3-indolyl-d-galactoside; 25 μL) were spread on the plate surface for blue/white colony selection. In addition to this selection, colonies accumulating reddish brown compounds were selected as BNP degraders according to the results of BNP-degrading *Sphingomonas* sp. NP5 (31). Selected transformants were examined to confirm the presence of the cloned sequence in the plasmid. In the assay of the BNP isomer degradation profile, *E. coli* JM 109 transformants were grown in LB/Amp liquid medium at 37°C for 24 h. *E. coli* cells were collected by centrifugation, suspended in fresh LB/Amp at OD_600_ of 0.5, and distributed into a series of sterile, glass test tubes (Φ13 mm by 100 mm) in 2-mL portions. After 10 μL of BNP stock solution (5 g L^−1^) in acetone was added to each of the tubes (at a final concentration of 25 mg L^−1^), the cultures were incubated at 37°C with gentle shaking. Triplicate cultures were assayed for the residual BNP before and after 24 h of incubation. A 2-mL culture was mixed with 1 mL of salting out solution (0.5 M HCl saturated with NaCl) (6) and 2 mL of hexane containing 10 mg L^−1^ 4-*tert*-OP (Sigma-Aldrich, MO, USA) as the internal standard. After vigorous shaking, the resulting hexane layer was collected and used for BNP determination by gas chromatography–mass spectrometry (GC-MS). *E. coli* JM109 transformed with a plasmid containing no insert was grown as the reference to estimate the recovery of BNP from the grown culture. The recovery of each BNP group (mean±SD, *n*=3) relative to before the incubation ranged between 74±3% (BNP group 5) and 83±2% (BNP group 1) for the cultures grown for 24 h. The remaining BNP did not appear to be extractable by hexane, possibly due to its lipophilic property and tight binding to bacterial cells (1), and, thus, the calculation of the degradation rate was corrected by subtracting this non-specific disappearance of each BNP group. The GC-MS analysis was performed as previously described (15) on a GCMS-QP2010 system (Shimadzu, Kyoto, Japan) equipped with a fused silica capillary column (Petrocol^TM^ DH, 100 m×0.25 mm×0.5 μm film thickness; Supelco/Sigma-Aldrich, Bellefonte, PA, USA). The oven temperature program was as follows: 3 min at 60°C, 15°C min^−1^ to 190°C, hold for 50 min, 15°C min^−1^ to 300°C, and hold for 10 min isothermally. A one-way analysis of variance (ANOVA) followed by Tukey’s honestly significant difference (HSD) test were used to test for significant differences in the degradation ratios of BNP isomer groups for each transformant (13).

The genome of the strain DSM 16289^T^ consisted of one circular chromosome (SAMIE1; 4,221,764 bp, 64.8% G+C content, 3,614 coding sequences [CDSs]) with a coverage of 212-fold, and six circular plasmids, pSAMIE2 (sequence coverage, 195-fold; 212,396 bp, 63.3% G+C, 180 CDSs), pSAMIE3 (sequence coverage, 219-fold; 212,121 bp, 63.9% G+C, 178 CDSs), pSAMIE4 (sequence coverage, 261-fold; 55,670 bp, 61.4% G+C, 51 CDSs), pSAMIE5 (sequence coverage, 116-fold; 39,623 bp, 62.5% G+C, 35 CDSs), pSAMIE6 (sequence coverage, 551-fold; 22,371 bp, 62.9% G+C, 21 CDSs), and pSAMIE7 (sequence coverage, 1,016-fold; 9,977 bp, 60.9% G+C, 10 CDSs). The chromosome carried six rRNA operons and 54 tRNA genes. An *opdA* homolog (*opdA*_DSM16289_) was present in pSAMIE2, while no *nmoA* homolog was found in the chromosome or any of the plasmids. This was in contrast to strain JCM 10874^T^, which possessed not only an *opdA* homolog, but also the *nmoA* gene, identical to that of strain NP5 (26). The predicted amino acid sequence (535 amino acids) from *opdA*_DSM16289_ showed 99.4% amino acid sequence identity to that from the *opdA* of strain PWE1 (*opdA*_PWE1_) and 99.8, 99.4, and 99.1% identities to those from the *opdA* homologs of strain JCM 10874^T^ (*opdA*_JCM10874_), strain Bayram (*opdA*_Bayram_), and strain TTNP 3 (*opdA*_TTNP3_), respectively. As described by Takeo *et al.* (31), the *opdA*_DSM16289_-coded protein also showed lower identity (83.6%) to the *nmoA*-coded protein (Supplementary Fig. S1). A phylogenetic tree constructed from the predicted protein sequences of the known *opdA*s and *nmoA*s is shown in Supplementary Fig. S2. A gene cluster (7,939 bp; *hqdR*, *hqdA*, *hqdB*, *orf1*, *orf2*, *hqdC*, *hqdD*, *hqdE*, and *hqdF*) encoding a hydroquinone catabolic pathway was found in pSAMIE4. The amino acid sequences predicted from the contiguous genes excluding *orf1* and *orf2* showed complete identity to those predicted from the gene cluster found in strain TTNP3 (17, 19).

To examine the effects of sequence differences among *opdA*_DSM16289_, *opdA*_JCM10874_, and *nmoA*_JCM10874_ on substrate specificity, *E. coli* JM109 strains harboring pSA*opdA*, pSC*opdA*, and pSC*nmoA* were grown in the presence of 25 mg L^−1^ BNP, and the degradation profile was analyzed by GC-MS. All the transformed strains degraded BNP group 1 isomers more efficiently than with any other BNP group: the percent degradation rates of the BNP group 1 by the transformants harboring pSA*opdA*, pSC*opdA*, and pSC*nmoA* were 34, 62, and 48%, respectively. To compare the degradation degrees of BNP isomer groups, we calculated the normalized ratio (called the degradation ratio) of the degradation rate for each BNP isomer group to that for isomer group 1. *E. coli* JM109 harboring pSA*opdA* showed lower degradation ratios against BNP isomer groups 3, 4, and 5 (degradation ratios, 0.33, 0.24, and 0.20, respectively), which was consistent with our previous findings obtained using the original *Sphingobium* strains (15). In the case of *E. coli* JM109 harboring pSC*opdA*, the degradation ratios for BNP groups 3, 4, and 5 were also lower: 0.65, 0.44, and 0.46, respectively. In contrast, *E. coli* JM109 harboring pSC*nmoA* showed less preference to any BNP isomer group: degradation ratios, 1.0, 0.77, and 0.62 for BNP isomer groups 3, 4, and 5, respectively.

The predicted amino acid sequence of *opdA*_DSM16289_ differed from that of *opdA*_JCM10874_ by only one amino acid (F241V), while it differed from those of *opdA*_PWE1_ and *opdA*_Bayram_ by three amino acids (M119L, K205Q, and F241A) and that of *opdA*_TTNP3_ by five amino acids (G58A, L87S, M119L, K205E, and F241C) (Table 2). Based on this result, the amino acid residue at position 241 appears to be important for substrate specificity. Porter *et al.* (28) proposed a homology model of the predicted structure of OpdA and suggested that the amino acid residues at positions 58 and 241 were located in the vicinity of the substrate-binding pocket, which was also suggested by our protein structure prediction using SWISS-MODEL with the OpdA-related flavoprotein *p*-hydroxybenzoate hydroxylase (EC 1.14.13.2) (12, 35). This was also the case for the *nmoA*-coded proteins (531 amino acids) of strains NP5 and JCM 10874^T^: G54 and A237, respectively. As summarized in Table 2, the change at residue 58 was only found in OpdA_TTNP3_ and was a conservative change from glycine to alanine. Therefore, the change at residue 241 near the substrate-binding pocket may be assumed to partly influence substrate specificity. This appears to be supported by our results showing that strain DSM 16289^T^ had the *opdA* homolog with narrow range substrate degradation activity (degradation ratio; 0.20 to 0.33 for BNP groups 3–5) and strain JCM 10874^T^ had *nmoA* (degradation ratio; 0.62 to 1.02 for BNP groups 3–5) and the *opdA* homolog (degradation ratio; 0.44 to 0.65 for BNP groups 3–5) with more versatile substrate specificity.

In conclusion, the present results suggest that strains JCM 10874^T^ (26) and DSM 16289^T^ degrade BNP by the concerted effects of different plasmids separately harboring the initial oxygenation gene (*opdA*) and hydroquinone catabolic pathway genes. The different preference of the two strains for BNP isomers may partly result from *nmoA* in strain DSM 10874^T^ and the different structure of the substrate-binding pockets in their OpdAs. To further confirm this, structure-activity analyses of OpdAs from the other BNP-degrading *Sphingomonas*/*Sphingobium* strains are needed.

## Accession number

The complete genome sequences of *S. amiense* DSM 16289^T^ were deposited into the DDBJ/EMBL/GenBank databases under the accession numbers AP018664 to AP018670 and were linked to the BioProject with the accession number PRJDB6948.

## Supplementary Material



## Figures and Tables

**Table 1:** Degradation profiles of branched nonylphenol isomer groups by *Escherichia coli* JM109 strains harboring pSA*opdA*, pSC*opdA*, and pSC*nmoA*, constructed from *Sphingobium amiense* DSM 16289^T^ (pSA*opdA*) and *Sphingobium cloacae* JCM 10874^T^ (pSC*opdA* and pSC*nmoA*).

No.	Representative structure of BNP isomer group	Degradation ratio/*E. coli* transformant with		

		pSA*opdA*	pSC*opdA*	pSC*nmoA*
1	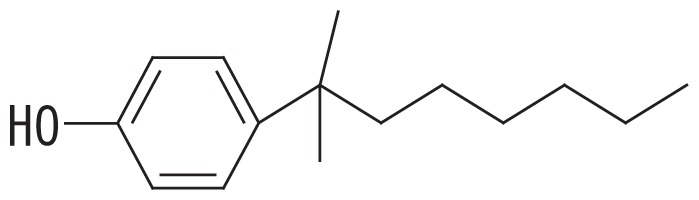	1.00 (0.10)^a^	1.00 (0.04)^a^	1.00 (0.04)^a^
2	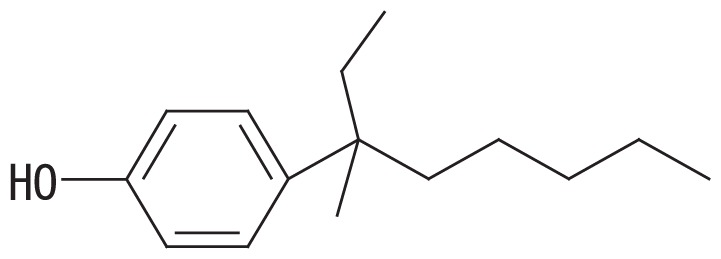	0.88 (0.14)^a^	0.73 (0.06)^b^	0.86 (0.04)^ab^
3	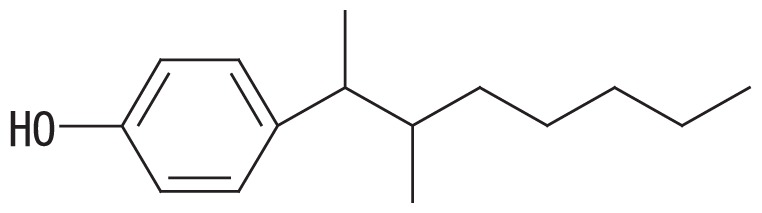	0.33 (0.08)^b^	0.65 (0.07)^b^	1.02 (0.08)^a^
4	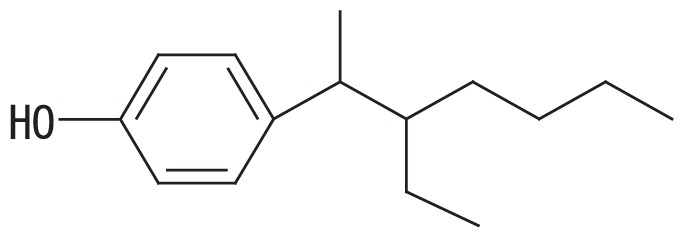	0.24 (0.03)^b^	0.44 (0.02)^c^	0.77 (0.19)^ab^
5	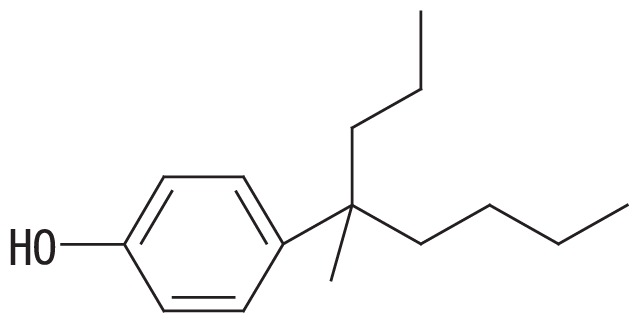	0.20 (0.14)^b^	0.46 (0.06)^c^	0.62 (0.07)^b^

Fourteen BNP isomers were separated into five groups by the GC-MS analysis: group 1, isomers with the α-dimethyl structure; group 2, isomers with the α-methyl, α-ethyl structure; group 3, isomers with the α-methyl, β-methyl structure; group 4, isomers with the α-methyl, β-ethyl structure; and group 5, isomers with the α-methyl, α-propyl structure (15). The degradation ratio is defined as the normalized ratio of the degradation rate for each BNP isomer group to that for isomer group 1 after a 24-h incubation. These values are the means of triplicate runs and standard deviation values are shown in the brackets. Mean values in the same column with common letters are not significantly different from each other according to a one-way ANOVA with Tukey’s HSD test, *p*<0.05. The percent degradation rates of isomer group 1 by the transformants harboring pSA*opdA*, pSC*opdA*, and pSC*nmoA* were 34, 62, and 48%, respectively. The commercial BNP product used in this study, in groups 1 to 5, contained 56.2, 28.0, 7.2, 3.0, and 5.6% BNP, respectively.

**Table 2:** Differences in amino acid residues predicted from *opdA* and *nmoA*, encoding enzymes for branched nonylphenol degradation.

Protein/Strain (Accession number)	Amino acid residue at the position of OpdA (NmoA)				

	58 (54)	87 (83)	119 (115)	205 (201)	241 (237)
OpdA					
*Sphingobium amiense* DSM 16289^T^ (BBE00139)	G	L	M	K	F
*Sphingobium cloacae* JCM 10874^T^ (BAV66737)	G	L	M	K	V
*Sphingomonas* sp. PWE1 (ABU98341)	G	L	L	Q	A
*Sphingobium xenophagum* Bayram (ACI12953)	G	L	L	Q	A
*Sphingomonas* sp. TTNP3 (ACI12952)	A	S	L	E	C
NmoA					
*Sphingobium cloacae* JCM 10874^T^ (BAV66828)	G	L	L	E	A
*Sphingomonas* sp. NP5 (BAI43691/BAI43694)	G	L	L	E	A
