# Supplementary material for: Comparative Analysis of the Genetic Basis of Branched Nonylphenol Degradation by Sphingobium amiense DSM 16289T and Sphingobium cloacae JCM 10874T
Source: Microbes Environ. 2018 Dec 5;33(4):450–4. doi: 10.1264/jsme2.ME18077 (PMC6308004; doi:10.1264/jsme2.ME18077)
Supplement: Supplementary file 1 [file 33_450_s1.pdf]

```
1
MENEMTTEPGDRASQFDVAIVGCGPVGALLANLLKQYGHKVAVLEREPDIFYAPRGMGFD
----MTAELTNQAAQFDVAIVGCGPVGALLANFLKQYGHKVAILDRELDVFYAPRGMGFD
    **:*  ::*:******************:*********:*:** *:**********
61
DESTRIMQSAGILDRLKAEGHIYQADLELIDRSGKRLGGFDRRSVGEDLLSGLHGHRHMT
DESTRIMQSVGILDRLKAEGHIYQADLELIDRNGKRLGGFDRRSVGEDLLSGLHGHRHLT
*********.**********************.*************************:*
121
LFHQPSLEATLREEFATGENAATAYFNHEVTGITDQGDRVELNCKDRATDEEHSLIAKYV
LFHQPSLEAILREEFSVGENAASSFFYHEVTEINDLGEQVELRCKNRATNEDFTISASYV
********* *****:.*****:::* **** *.* *::***.**:***:*:.:: *.**
181
VGCDGARSTVRKTMNVPRIDLKYTKKYLVVDAIVDDPVYFRTMIPQGGYILLDGKEAGVL
VGCDGARSIVRKTMSVPRVDLKYTERYLVVDAIVDDPVYFRTRIPQGGYILHDGKEAGVL
******** *****.***:*****::**************** ******** ********
241
VKGLHGHVRFDFLQHSETIGQELKTDEDYQKAARDLIRSRGFDPENFRVIRSVSYTFHAG
AKGLHGHVRFDFLQHSEIVGAELKNEKDYQDAARALIESRGFEPENFRVIRSVSYTFYAG
.**************** :* ***.::***.*** **.****:**************:**
301
MPSKWRVGRLMVAGDAAHLTPPWSGQGLNMGVRDAANLSFKLNLALRGKSSDRILDTYDE
MPSRWRVGRLLVAGDAAHLTPPWSGQGLNMGIRDAANLSFKLSLVLNKKTSDRILDTYDD
***:******:********************:**********.*.*. *:*********:
361
ERRPQSLETIQAAVDMGIRMQNTSPLQIGLRNLAYALSRKSKFVNRLLFKNWIRKPSYKS
ERRPQSLETIQAAVDMGIRMQSTSPLQIGLRNFVYALSRSSKFVNRLLFRNWIRKPGFKS
*********************.**********:.*****.*********:******.:**
421
GLLGLQHRLSGGPMFQPWVETAEGKRVRMDDLIGLNFALISTDSPTGPEVRQFVSELGGV
GLIGLQHRLSGTPMFQPWVQTPDGERRRMDDLIGLRFALISTDSPTGPEVTDFVRSLGGV
**:******** *******:*.:*:* ********.************** :** .****
481
VLKLDCDFFDPSETVCKWYDEHRINAVLLRPDRVIYDAGRDGQALCRSLLAELRK
VLKLDCDFFDPGETVCKWYDKNRINAVLLRPDRVIYDAGRDGRALCRSLLSELRK
***********.********::********************:*******:****
```

Supplementary Fig. S1. Alignment of amino acid sequences predicted from *opdA* (upper sequence) and *nmoA* (lower sequence) of *Sphingobium cloacae* DSM 10874^{T}.

*, identical; :, strong similarity; ., weak similarity.

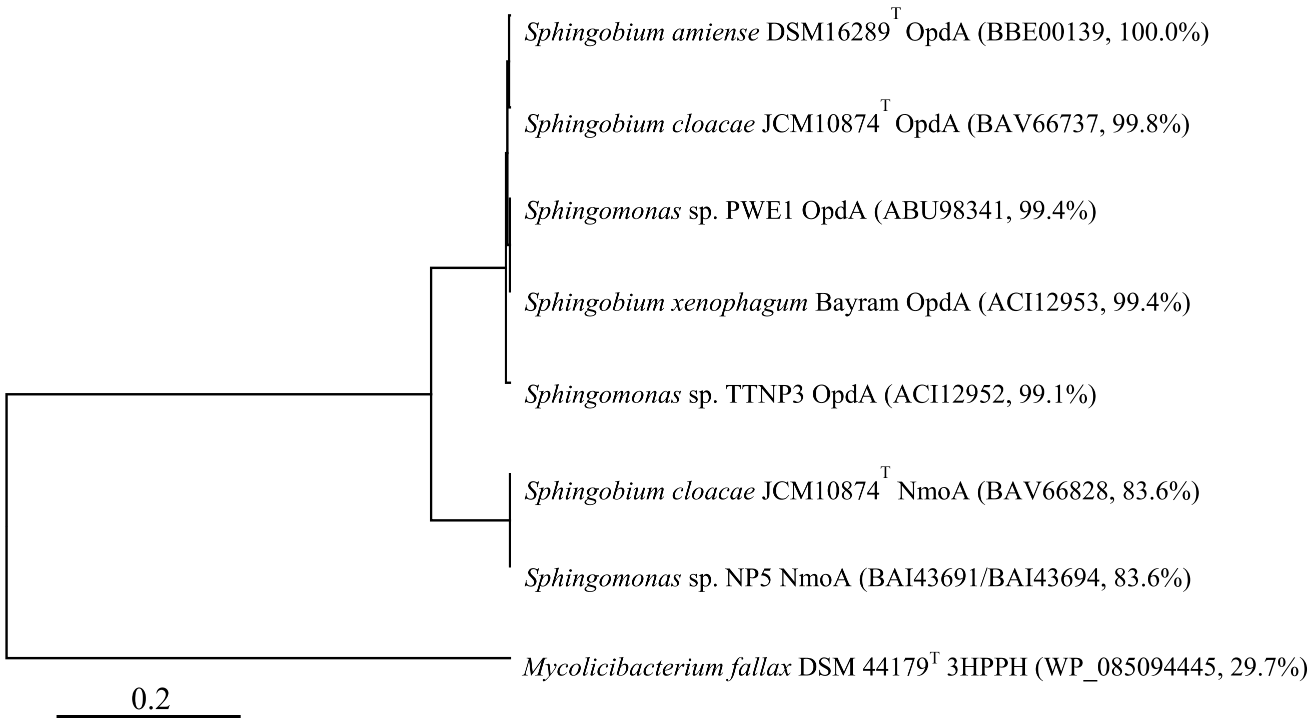

Supplementary Fig. S2. Phylogenetic tree based on amino acid sequences predicted from the known genes *opdA* and *nmoA*, involved in branched nonylphenol degradation. The tree was constructed by the UPGMA method with bifunctional 3-(3-hydroxy-phenyl) propionate/3-hydroxycinnamic acid hydroxylase (3HPPH) of *Mycolicibacterium fallax* DSM 44179^{T} as the outgroup. Accession numbers and amino acid sequence identity with the OpdA homolog of strain DSM 16289^{T} are shown in parentheses.
